# The Poor in Towns

**Published:** 1887-05-14

**Authors:** 


### THE POOR IN TOWNS.

Those readers who followed our advice and took a little holiday in the spring, ought to take a special interest in the welfare of the less favoured, who for want of means have been prevented from exchanging the slavery of the town for the freedom of the country. To all who love the country, who realise the value and rejuvenating properties of its fresh air, the condition of the occupants of tenement-houses, and especially of the children in poor town neighbourhoods, must awaken the deepest sympathy, and a fervent desire to support any movement which will lighten the lot of those condemned to live under such disadvantageous conditions. "One touch of nature makes the whole world kin," and the emotions we refer to are the common property of no individual or nation, but the care of the poor is regarded as a sacred trust by the thoughtful citizen all the world over. The American, with his quick perception, has anticipated the Englishman in this matter. The frightful sufferings and enormous death-rate of the children in the crowded and poorer portions of New York City in the hot season sent a thrill of horror through the wealthier quarters, and resulted in the establishment of a system of convalescent cottages, to which poor town residents might be sent for a good holiday in the summer time.

Madame Batthyany, of Eaglehurst, was the first lady to try this system in England. Her scheme is to get a certain number of children from the poor overcrowded portions of London, say six, with one grown-up woman, either a sister, or a poor overworked drudge, or sempstress, or a mother, to come to the country for a week. By changing the visitors every week during the summer months, one hundred poor people and children can easily be placed within reach of the pleasures and advantages of a week in the country. The object is to arrange that every child shall have one week's holiday every year, that it shall be placed under conditions of happiness and health which will give it a memory and a hope. The idea is to give these poor children a run in the fields, or by the sea-shore, to let them live only a little better than they do at home, to make them feel themselves free of school, and to place them in communion with nature and the rest of humanity.

Every village should have a few rooms for such guests, and all the villagers can aid the scheme. The farmer's wife can give a little food, milk, eggs, butter, and the like; the rich can help by giving money to pay for the hire of rooms, or a cottage specially devoted to the purpose, and to defray the cost of railway and other travelling expenses; the young ladies can turn their needles to account by repairing, mending, or making clothes for the little strangers; all can give these poor little waifs and strays a hearty welcome and a cheering word. We know one village, Gosfield in Essex, where Miss Courtauld and Mrs. Elliot have introduced this common link of humanity between the occupants of the crowded city tenements, and the farm-house and village cottage, with such excellent results that the people in the village welcome their summer visitors, and look forward to their arrival with the keenest interest. Similarly, the Rev. Miles Atkinson, one of the curates of that most useful and earnest of men, Mr. Barnett, Vicar of St. Jude's, Whitechapel, can testify, from the town resident's point of view, that this system is the means of doing all kinds of good to both parents and children. There is nothing pauperising about it, and parents gladly defray the railway-fare of their children, whilst they thankfully accept the holiday as a gift, and not as charity. We shall have more to say about Whitechapel experiences on another occasion, and shall welcome an account of the experiences and suggestions of Mr. Atkinson, Mr. Barnett, Miss Courtauld, or of anyone who either desires to introduce the system or who has tried it, and will thus help the cause.

One word in conclusion to the fathers and mothers of children who have the good fortune to be able to take a holiday in the country or at the sea. Think of the lot of the children of the poor in the crowded tenement-houses of the busy city in which you live; contrast it with your own lot or with that of your children; and when you return from your own holiday, rejoice at the improved health and appearance of your family or yourselves, and reflect how much more necessary is a change of air for the neighbouring poor. One sovereign will enable one child to have a holiday in the country for a month, or four children for one week each. Who will not give a sovereign in such a cause, remembering that the tenement-house children, who by such bounty return home better in health and with renewed strength and spirits, must thereby be made better citizens. The children bring back to the town little stocks of news, little trifles which youngsters love—shells, pebbles, a flower-pot with a real plant in it, and a hundred odds and ends of things that will amuse them throughout the winter, and which big houses at present throw away as too insignificant to be given to their own poor. Each child will have new subjects of interest, and it will give its playmates the history of its week of happiness, to their mutual advantage. These little ones will look forward to their next holiday, will make plans not all sordid, and have ideas not wholly selfish. All will learn how to work themselves, and will see how others labour. Brought into contact with a new world and with new people, they will also see that woods and water and sky and sunshine are pleasant realities. Returning home, they will carry with them a current of fresh outer air back to their pent-up city life, to the great gain of all their little circle.

The bare recital of such momentous results from such small expenditure warms one's heart. We desire to give this feeling practical value by opening our columns for the purpose of bringing together those in the country who will try the system, and those in the town who know not how to help the little ones languishing, ay, dying, for the lack of fresh air and change during the hottest portion of each year. Let the country clergy and squires, and the town clergy and district visitors, communicate with us, and we will gladly do our part to facilitate necessary arrangements, and so to benefit great numbers of poor children. Any contributions in money from those who have means, but neither time nor opportunity to help in other ways, will be gratefully received and duly acknowledged.

